# Imaging EGFR and HER3 through ^89^Zr-labeled MEHD7945A (Duligotuzumab)

**DOI:** 10.1038/s41598-018-27454-6

**Published:** 2018-06-13

**Authors:** Brooke N. McKnight, Akhila N. W. Kuda-Wedagedara, Kuntal K. Sevak, Dalya Abdel-Atti, Wendy N. Wiesend, Anson Ku, Dakshnamurthy Selvakumar, Sean D. Carlin, Jason S. Lewis, Nerissa T. Viola-Villegas

**Affiliations:** 1 Department of Oncology, Karmanos Cancer Institute, 4100 John R. Street, Detroit, MI 48201 USA; 2 Department of Radiology, Memorial Sloan Kettering Cancer Center, 1275 York Avenue, New York, NY 10065 USA; 3 Department of Anatomic Pathology, Beaumont Hospital, 3601 West 13 Mile Road, Royal Oak, MI 48073 USA; 4 Program in Molecular Pharmacology, Memorial Sloan Kettering Cancer Center, 1275 York Avenue, New York, NY 10065 USA; 5 Weill Cornell Medical College, 1300 York Avenue, New York, NY 10065 USA

## Abstract

Tumor resistance to treatment paved the way toward the development of single agent drugs that target multiple molecular signatures amplified within the malignancy. The discovered crosstalk between EGFR and HER3 as well as the role of HER3 in mediating EGFR resistance made these two receptor tyrosine kinases attractive targets. MEHD7945A or duligotuzumab is a single immunotherapy agent that dually targets both molecular signatures. In this study, a positron emission tomography (PET) companion diagnostic to MEHD7945A is reported and evaluated in pancreatic cancer. Tumor accretion and whole body pharmacokinetics of ^89^Zr-MEHD7945A were established. Specificity of the probe for EGFR and/or HER3 was further examined.

## Introduction

The research landscape for the human epidermal growth factor family of receptors has expanded with the discovery of their additional diverse roles in cancer. Extensive study of their function in aiding carcinogenesis and resistance to therapy is characterized and described in a number of reports^1–3^. In pancreatic cancer, the epidermal growth factor receptor (EGFR) is expressed in 30–90% of patients with pancreatic ductal adenocarcinoma (PDAC)^4–6^, marking aggressive disease with poor survival rates. EGFR has notably contributed to its early carcinogenesis from normal pancreatic epithelia, which transitions to neoplasms of pancreatic intraepithelial (PanIN) and finally, forming PDAC^7^.

Receptor tyrosine kinases are implicated in resistance to treatment with their blockade stimulating compensatory pathways to rescue signaling activity. Recent studies reported that antagonism of EGFR resulted in the induction of other compensatory pathways such as the human epidermal receptor 3 (HER3) receptor^8–10^. HER3 amplification in solid tumors is associated with poor survival and resistance to therapy^11^. For example, cetuximab treatment demonstrated increased HER3 in colon^12^, head and neck^13^ and triple negative breast cancer^14^. In PDAC, HER3 is the preferred dimerization partner of EGFR^15^ with its concomitant activation rendering this malignancy impervious to EGFR and HER2 targeted therapy^5^. Furthermore, EGFR and HER3 are highly expressed in PDAC, marking this aggressive disease with poor survival rates^5,6^.

With this perspective, combinatorial treatment strategies emerged to simultaneously target both the primary tumor’s molecular signature (e.g. EGFR) as well as the signaling mechanism likely to develop (e.g. HER3) upon resistance to first line therapy^16^. MEHD7945A or duligotuzumab, is a single agent fully human IgG1 monoclonal antibody (mAb) that targets both EGFR (K_D_ ~ 1.9 nM) and HER3 (K_D_ ~ 0.4 nM)^17^. It was developed to improve treatment response of solid tumors confounded with HER3-mediated resistance to EGFR-targeted treatment^17^. It is also efficacious in tumors refractory to both radiation and prolonged EGFR-specific treatment^18,19^. Importantly, it is safely tolerated by patients with locally advanced or metastatic epithelial cancers with no dose-limiting toxicities^20^. Partial response rates have been achieved in patients with cetuximab-refractory and prior chemo radiation squamous cell carcinoma of the head and neck (SCCHN)^20^.

A companion diagnostic to MEHD7945A is critical for patient selection. In this study, we report the development of ^89^Zr (t_1/2_ = 3.27 d) labeled MEHD7945A (^89^Zr-MEHD7945A) and an evaluation of its pharmacological properties in PDAC by evaluating *in vivo* spatial distribution of the tracer against regional localization of EGFR and HER3 in Kras wild-type (BxPC-3) and mutant (AsPC-1) pancreatic cancer. We further investigated its specificity to EGFR and/or HER3 through *in vitro*, *in vivo a*nd *ex vivo* competitive blocking studies. Shifts in EGFR and HER3 expression during these blocking assays were measured by the radiotracer and further validated through immunoblots, flow cytometry and immunohistochemistry.

## Results

### Characterization of ^89^Zr-MEHD7945A

The labeling of MEHD7945A with ^89^Zr was straightforward. Radiolabeling yields of >95% were obtained with >99% purity after purification. A specific activity of 4.53 ± 0.65 mCi/mg (25.5 ± 3.7 MBq/nmol) was established. The labeled protein retained its immunoreactivity toward both EGFR and HER3 with 74 ± 0.5% (n = 3) retention, which is within range of acceptable immunoreactivities (>60%) for clinical use^21–25^. ^89^Zr-MEHD7945A remains moderately intact >94% in both saline and 1:1 human serum:saline, over a 120 h incubation period at 37 °C (Supplementary Fig. S1).

### EGFR and HER3 expression in established pancreatic cancer cells

Among the three pancreatic cell lines, AsPC-1 (Supplementary Fig. S2A) displayed the highest EGFR and HER3 staining with ~85% of the cell population co-expressing both receptors. BxPC-3 (Supplementary Fig. S2B) demonstrated approximately ~74% of the cell population staining for both receptors. A very low level of Mia PACA2 (Supplementary Fig. S2C) cells co-express both receptors (0.42%). Western blots demonstrated relatively equal expression of EGFR between AsPC-1 and BxPC-3 cell lines, with almost no EGFR expression in Mia PACA2 (Supplementary Fig. S2D). The HER3 order of expression for the three pancreatic cells are as follows: AsPC-1 > BxPC-3 > Mia PACA2.

### *In vitro* internalization studies

**Figure 1:**
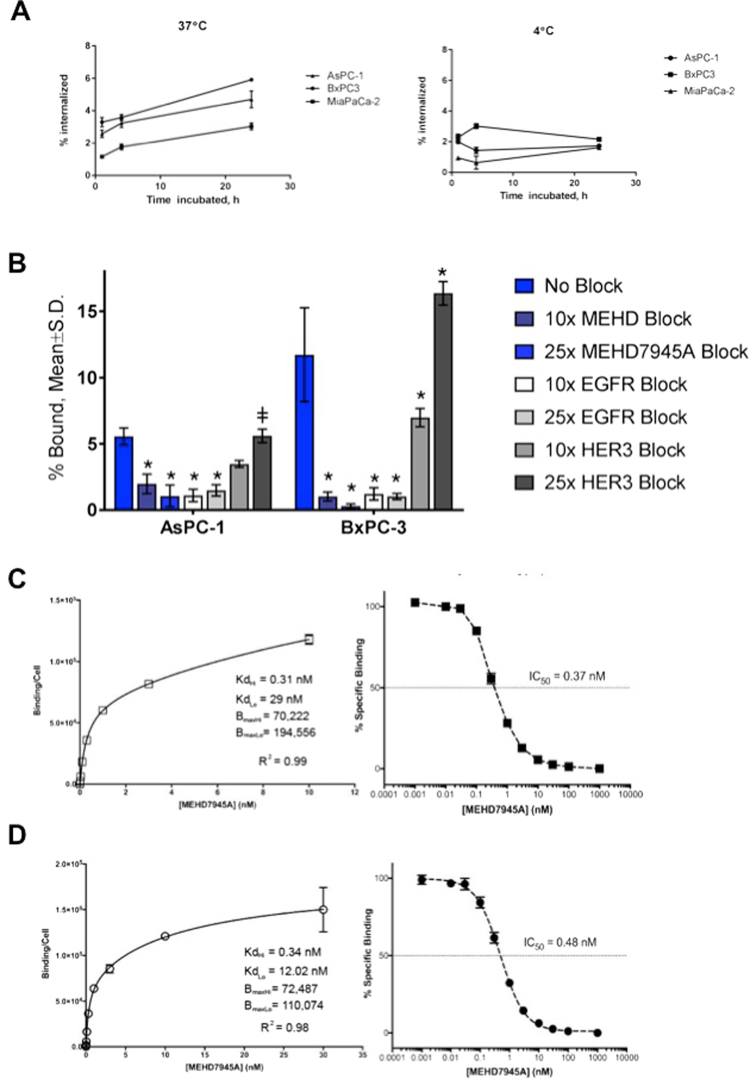
*In vitro* characterization. ^89^Zr-MEHD7945A demonstrated higher internalization rates in BxPC-3 and AsPC-1 at 37 °C (left) compared to the EGFR/HER3-negative Mia PACA2 pancreatic cancer cell line. ^89^Zr-MEHD7945A showed a decrease in internalization at 4 °C (right) in all cell lines. (**A**) ^89^Zr-MEHD7945A demonstrated successful blocking with cold MEHD7945A and cetuximab at 10× and 25× doses. Blocking with DL3.6b at 10× lowered the uptake of the probe; binding was sustained at 25× dose of the anti-HER3 mAb. In both AsPC-1 and BxPC-3. (**B**) Non-linear regression analysis determined two sets of K_D_ and B_max_ for AsPC-1 with an. (**C**) The K_D_ and B_max_ values for BxPC-3 (**D**) are within the same range as the established values in AsPC-1 with an IC_50_ ~ 0.37 nM. (*Denote p < 0.01, ^ǂ^denote p < 0.05, compared to no block).

Internalization of ^89^Zr-MEHD7945A in all cell lines was conducted at 37 °C (Fig. 1A, left). BxPC-3 displayed the highest uptake from 3.29 ± 0.28% at 1 h to almost a two-fold increase at 24 h with 5.91 ± 0.05% of the tracer internalized. In AsPC-1, the tracer was steadily internalized over time (2.58 ± 0.23% at 1 h, 3.23 ± 0.26% at 4 h and 4.70 ± 0.52% at 24 h) while the negative control cell line, Mia PACA2 demonstrated lower uptake across all time points (1.15 ± 0.06% at 1 h, 1.76 ± 0.17% at 4 h and 3.04 ± 0.21% at 24 h respectively). Significantly lower accumulation was observed in all cell lines at 4 °C, supporting an endocytotic mechanism of internalization (Fig. 1A, right).

### *In vitro* competitive inhibition studies

In AsPC-1 cells, (Fig. 1B and Supplementary Table S1) co-administration of ^89^Zr-MEHD7945A and unlabeled MEHD7945A at an excess of 10- and 25-fold for 1 h reduced uptake (1.97 ± 0.74%, p < 0.0001 and 1.06 ± 0.82%, p < 0.0001, respectively) compared to unblocked cells (5.55 ± 0.64%). Blocking with the anti-EGFR antibody, cetuximab, which binds to the same EGFR epitope as MEHD7945A, also significantly reduced the radiotracer uptake (1.09 ± 0.47% at 10× and 1.47 ± 0.42% at 25× blocking doses, p < 0.0001). Competitive inhibition of the tracer with the anti-HER3 antibody DL3.6b with a 10-fold excess decreased tracer binding from 5.55 ± 0.64% to 3.47 ± 0.26% (p = 0.0194), whereas treatment with 25-fold excess of DL3.6b showed no significant difference with 5.60 ± 0.51% total bound compared to control. External validation through flow cytometry was performed on separate groups of AsPC-1 cells exposed for 48 h to 25-fold higher cold doses of MEHD7945A, cetuximab and DL3.6b (Supplementary Fig. S3A and Supplementary Table S1). Control untreated cells had a median fluorescent intensity (MFI) of 340.3 ± 32.8 for EGFR-expressing cells and 79.6 ± 11.5 for HER3-expressing cells. Blocking with MEHD7945A significantly reduced EGFR expression compared to control (32.6 ± 2.8 MFI, p < 0.0001) but did not significantly reduce cells bearing HER3 expression (57.3 ± 3.7 MFI, p = 0.096). Blocking with cetuximab significantly depleted EGFR expression (0.1 ± 0.9 MFI, p = 0.0001) but not HER3 (80.4 ± 6.9 MFI). Treatment with DL3.6b did not change EGFR expression (336.0 ± 3.6) but significantly attenuated total HER3 (48.2 ± 4.9 MFI, p = 0.0128).

In BxPC-3 cells, normal uptake of the radiotracer was measured at 11.72 ± 3.54% (Fig. 1B and Supplementary Table S1). Binding was significantly reduced when co-administered with MEHD7945A (10-fold: 1.02 ± 0.34%, p = 0.0001; 25-fold: 0.29 ± 0.16%, p = 0.0001) and cetuximab (10-fold: 1.21 ± 0.46%, p = 0.0001; 25-fold: 1.02 ± 0.23%, p = 0.0001). Competition with 10-fold DL3.6b lowered radiotracer accumulation to 6.98 ± 0.69% (p = 0.0001). Interestingly, radiotracer uptake was not diminished at 25-fold excess DL3.6b with 16.36 ± 0.88% binding (p = 0.0001). From the flow cytometry analysis, control cells exhibited 307.7 ± 11.2 MFI and 127.0 ± 3.6 MFI for EGFR and HER3 respectively. MEHD7945A-blocked cells decreased EGFR expression (182.7 ± 28.4 MFI, p = 0.0001) and HER3 (95.4 ± 7.7 MFI, p = 0.0123). Exposure to cetuximab mitigated EGFR expression (3.3 ± 0.8 MFI, p = 0.0001) whereas HER3 abundance remained unchanged (130.3 ± 4.0 MFI). Incubation with DL3.6b decreased HER3 (63.3 ± 8.2 MFI, p = 0.0001) while EGFR levels remained similar to unblocked control (284.0 ± 13.1 MFI, p = 0.0739) (Supplementary Fig. S3 and Supplementary Table S1).

### *In vitro* binding affinity

Surface plasmon resonance (SPR) analysis of the dissociation constant (K_D_) of DFO-derivatized MEHD7945A vs. the unmodified mAb demonstrated relatively similar binding for HER3 (37 pM vs. 8.1 pM, respectively) and EGFR (3.4 nM vs. 1.9 nM respectively). Competitive binding of ^89^Zr-MEHD7945A with increasing concentrations of cold MEHD7945A was conducted in both BxPC-3 and AsPC-1 cells. In AsPC-1 (Fig. 1C, left), a K_DHi_ ~ 0.31 nM and K_DLo_ ~ 29.00 nM were achieved. Measured B_maxHi_ and B_maxLo_ values were ~1.94 × 10^5^ and ~7.02 × 10^4^ receptors sites were available. The IC_50_ was ~0.37 nM (Fig. 1C, right). From the plot in Fig. 1D (left), the dissociation constants K_DHi_ and K_DLo_ in BxPC-3 cells were determined to be 0.34 nM and 12.02 nM with a B_maxHi_ of ~7.25 × 10^4^ and B_maxLo_ of ~1.10 × 10^5^. The IC_50_ for ^89^Zr-MEHD7945A was observed to be ~0.48 nM in BxPC-3 (Fig. 1D, right).

### *In vivo* EGFR/HER3-PET Imaging

**Figure 2:**
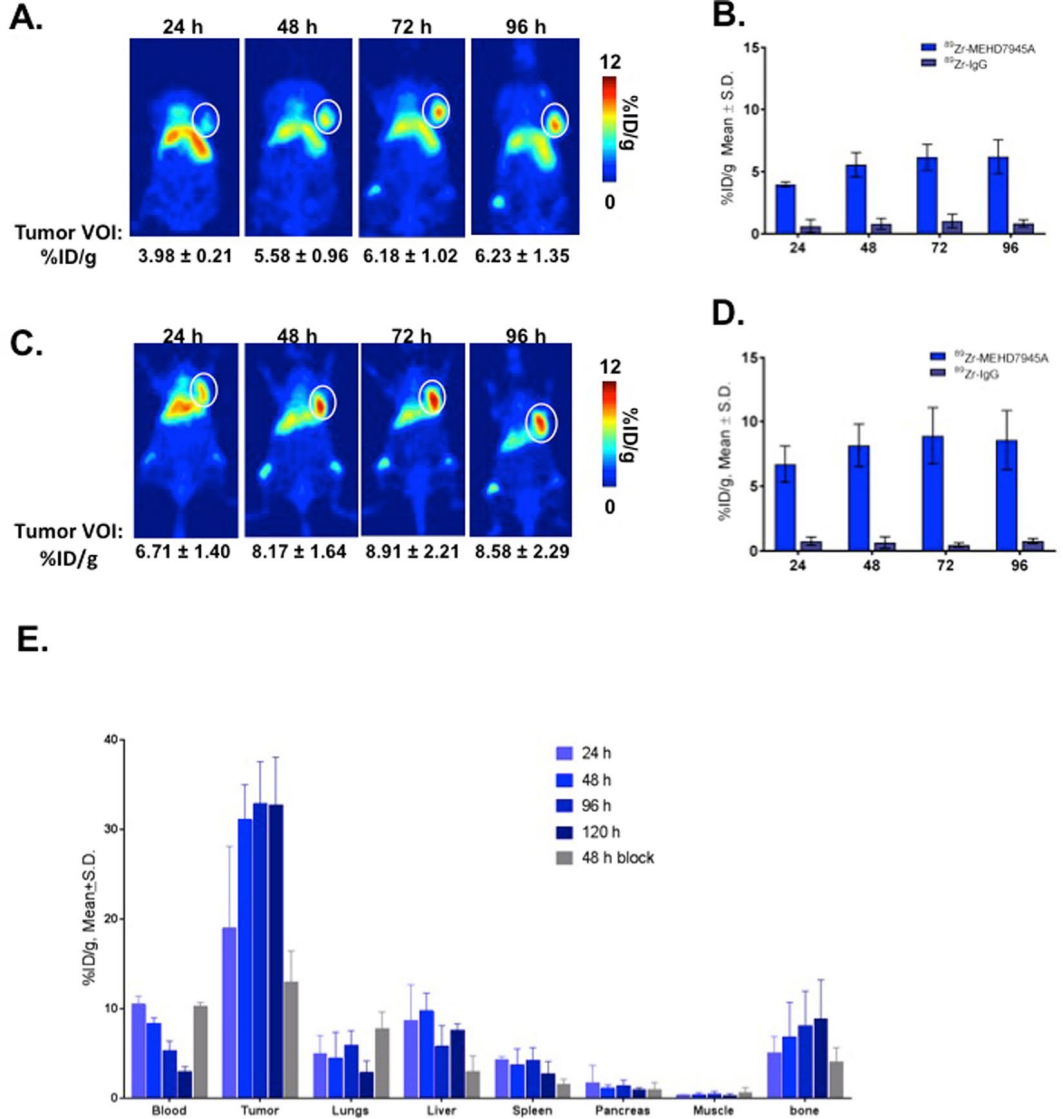
*In vivo* PET imaging. In AsPC-1 xenografts, tumor volumes-of-interest (VOI) expressed as % ID/g generated from the ^89^Zr-MEHD7945A PET scans exhibited uptake as early as 24 h p.i., peaking at 72 h p.i. and retained to as long as 96 h p.i. (**A**) ^89^Zr-IgG control PET scans showed minimal uptake within the tumor at all time points. (**B**) Similarly in BxPC-3 tumors, PET scans exhibited uptake at 24 h p.i. with a peak at 72 h p.i. (**C**) Non-specific tumor uptake using ^89^Zr-IgG in BxPC-3 xenografts showed nominal accumulation across all time points. (**D**) Whole body tissue distribution revealed high tumor tissue uptake of the tracer at 24 h p.i,, which plateaued at 48 h through 120 h p.i. in BxPC-3 xenografts. A competitive blocking study using unmodified MEHD7945A at 48 h p.i. displayed at least a two-fold decrease in tumor binding, indicative of the probe’s specificity. (**E**) Of note, normal pancreas demonstrated minimal non-specific binding on all time points, suggesting that an excellent signal-to-noise contrast can be achieved.

In Fig. 2A, cumulative tumor uptake of ^89^Zr-MEHD7945A was observed in the AsPC-1 xenograft with 3.98 ± 0.21 percent injected dose per gram of tissue (%ID/g) at 24 h post-injection (p.i.) with the maximum tumor-bound activity achieved at 72 h p.i. at 6.18 ± 1.0%ID/g. Retention of the radiotracer was observed to as far as 96 h p.i. (6.23 ± 1.35%ID/g). AsPC-1 tumors imaged with the non-specific isotype ^89^Zr-IgG had significantly less uptake (0.62 ± 0.53%ID/g at 24 h, 0.80 ± 0.44%ID/g at 48 h, 1.03 ± 0.55%ID/g at 72 h, and 0.83 ± 0.29%ID/g, p < 0.001) at all time points (Fig. 2B). Higher cumulative tumor uptake was observed in BxPC-3 xenografts with 6.72 ± 1.40%ID/g at 24 h p.i. (Fig. 2C). The tumor uptake peaked at 8.91 ± 2.1%ID/g at 72 h with observed retention at 96 h p.i. (8.56 ± 2.3%ID/g). Separate BxPC-3 tumors imaged with ^89^Zr-IgG showed significantly less uptake (for example, 0.75 ± 0.32%ID/g at 24 h p.i, 0.65 ± 0.46%ID/g at 48 h p.i. p < 0.001, Fig. 2D) for all time points.

### Whole Body Distribution of ^89^Zr-MEHD7945A

Tissue distribution of ^89^Zr-MEHD7945A was analyzed in BxPC-3 tumor-bearing mice at 24–120 h p.i. (Fig. 2E and Supplementary Table S2). Tumor uptake was detected at 24 h p.i. with 24.6 ± 6.7%ID/g. The accumulation reached 31.1 ± 3.9%ID/g at 48 h p.i. and plateaued over the later time points with 32.8 ± 4.7%ID/g at 96 h p.i. and 32.7 ± 5.3%ID.g at 120 h p.i. Tumor accumulation decreased by two-fold (13.0 ± 3.4%ID/g) at 48 h p.i when ^89^Zr-MEHD7845A was administered with an excess of unmodified antibody to compete and block the radiotracer for receptor sites. Uptake in normal pancreas was significantly lower with <2%ID/g across all time points. Other organs within close proximity to the pancreas demonstrated low non-specific accumulation. For example at 48 h p.i., non-targeted binding in the liver, the primary clearance route for most mAbs, was 2.5-fold less than tumor uptake at 9.7 ± 2%ID/g. At the same time points, tissue-uptake values for the spleen, stomach and gut were 3.7 ± 1.8%ID/g, 1.3 ± 0.7%ID/g and 1.2 ± 0.3%ID/g respectively. Bone accumulation was observed over time with 6.84 ± 3.85%ID/g at 48 h p.i., which plateaued at 8.86 ± 4.34%ID/g at 120 h p.i. Blocking with MEHD7945A at 48 h p.i. moderately lowered the bone uptake to 4.03 ± 1.60%ID/g but was statistically insignificant.

### ***Ex Vivo*** Autoradiography and immunohistochemistry (IHC)

**Figure 3:**
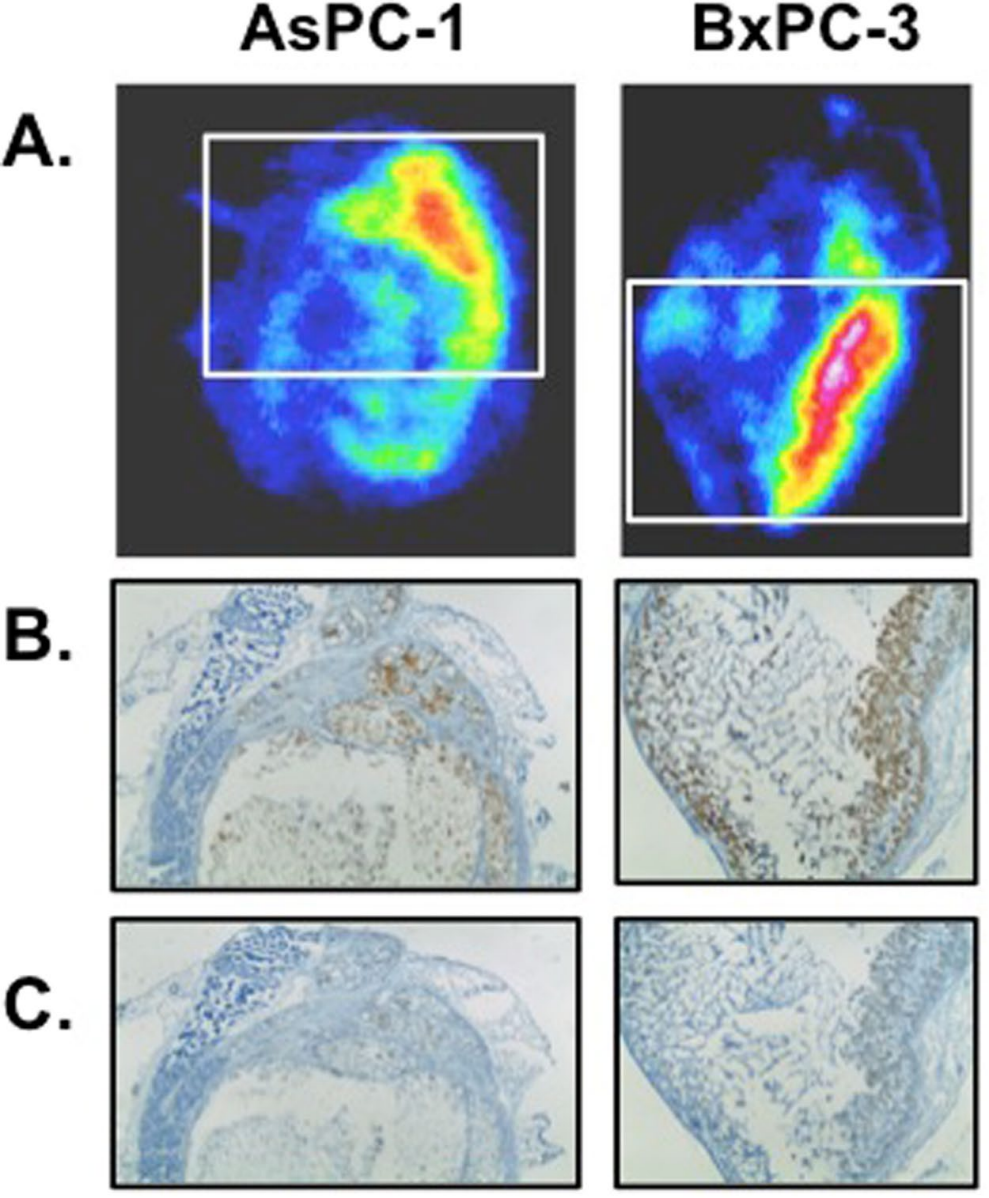
Autoradiography and histology. Autoradiographs (**A**) of excised AsPC-1 (left) and BxPC-3 (right) tumor sections depicted co-localization of the tracer in areas where EGFR (BxPC-3 3+, AsPC-1 2+) (**B**) and HER3 (BxPC-3 1+, AsPC-1 2+) (**C**) are expressed.

To understand the differences in tracer uptake between tumor models, spatial distribution of ^89^Zr-MEHD7945A was evaluated using digital autoradiography on AsPC-1 (Fig. 3A, left) and BxPC-3 tumors (Fig. 3A, right). Adjacent tumor sections of both AsPC-1 and BxPC-3 were evaluated for EGFR (Fig. 3B) and HER3 expression (Fig. 3C) by immunohistochemistry. Tissue viability and collagen content were qualitatively assessed via H&E staining (Supplementary Fig. S4A,B) and trichrome staining (Supplementary Fig. S4C), respectively. In both tumors, ^89^Zr-MEHD7945A accumulated in viable tumor regions with high EGFR expression (Fig. 3B). Staining of EGFR and HER3 showed strongest positivity on viable tumor cells. Although, the expression of EGFR in BxPC-3 and AsPC-1 tumor sections was similar, there was higher expression of HER3 in AsPC-1 when compared to BxPC-3. Areas with strong staining for HER3 co-localized with areas of high ^89^Zr-MEHD7945A registration (Fig. 3C). Taken together, the localization pattern indicates the dependence of ^89^Zr-MEHD7945A on both target expression and local pharmacokinetics. Collagen expression was qualitatively equivalent for both tumor sections. Looking at cell density, BxPC-3 tumors were observed to be two-fold more “cell dense” than AsPC-1 xenografts (258.8 ± 64.2 cells vs. 71.3 ± 14.9 cells, p = 0.0013, (Supplementary Fig. S4D).

### Assessment of EGFR and/or HER3 dual specificity of ^89^Zr-MEHD7945A

**Figure 4:**
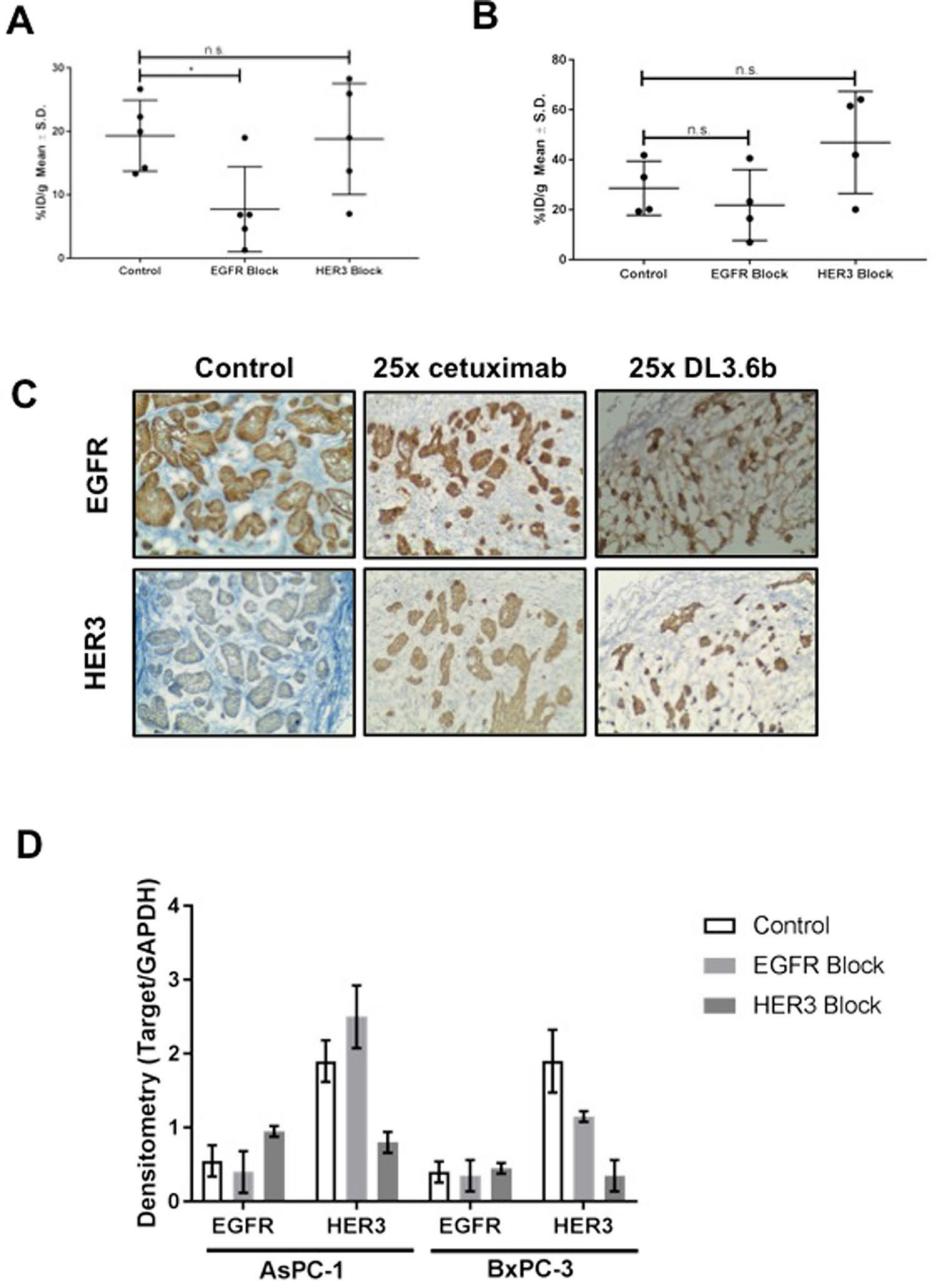
*In vivo* competitive inhibition. In AsPC-1 xenografts, blocking with cetuximab (EGFR block) showed an almost 2-fold decrease in ^89^Zr-MEHD7945A uptake, whereas blocking HER3 with DL3.6b did not change probe uptake. (**A**) In BxPC-3 xenografts, blocking with cetuximab (EGFR block) showed a slight decrease in ^89^Zr-MEHD7945A, whereas blocking with DL3.6b (HER3 block) showed a statistically significant, increase in probe accumulation. (**B**) IHC staining in BxPC-3 tumors blocked with 25× cetuximab (25x EGFR, left), 25x DL3.6b (25x HER3, middle) or left unblocked (right) were assessed by IHC for EGFR (top) and HER3 (bottom) expression, and showed an increase in EGFR and HER3 in both blocked cohorts. (**C**) Tumor sections depicted for IHC are shown in 100×. Densitometry analysis of western blots on tumor lysates (n = 2) from AsPC-1 (left) and BxPC-3 (right) that were untreated, exposed to EGFR-block with cetuximab and a HER3-block with DL3.6b. (**D**) Densitometry is shown as a ratio of target protein/loading control.

To determine whether ^89^Zr-MEHD7945A binds to either EGFR or HER3 alone or both, *in vivo* blocking studies were conducted. Blocking EGFR with cetuximab in AsPC-1 tumors (Fig. 4A and Supplementary Table S3) lowered the uptake of ^89^Zr-MEHD7945A by almost 3-fold (19.30 ± 5.60%ID/g vs. 7.72 ± 6.70%ID/g, p = 0.0457). In contrast, blocking the HER3 epitope with DL3.6b did not alter tumor accumulation of ^89^Zr-MEHD7945A (18.79 ± 8.75%ID/g).

In BxPC-3 xenografts (Fig. 4B and Supplementary Table S3), exposure to cetuximab showed no difference in ^89^Zr-MEHD7945A uptake between control (28.56 ± 10.81%ID/g) and cetuximab-blocked tumors (21.79 ± 14.19%ID/g, p = 0.39). Competition of the tracer with DL3.6b demonstrated an approximately two-fold increase in radiotracer accumulation of 46.89 ± 20.1%ID/g (p = 0.0021).

We next determined the underlying mechanism for the unexpected trend in tumor uptake of the blocked cohorts. Pathological analysis revealed an increase in EGFR (Fig. 4C, top) and HER3 (Fig. 4C, bottom) staining in both EGFR and HER3 blocked BxPC-3 tumors compared to control. From the western blots, cetuximab-treated AsPC-1 exhibited a two-fold decrease in total EGFR protein, coupled with a 1.3-fold increase in HER3 as shown by densitometry (Fig. 4D). With the DL3.6b blocked AsPC-1 tumors, a 2.25-fold increase in EGFR with a concomitant three-fold decrease in HER3 was displayed. Similar trends were observed in BxPC-3 wherein cetuximab-blocked groups displayed suppressed EGFR and HER3 (Fig. 4D). Saturation of the HER3 sites exhibited a moderate increase in EGFR expression, coupled with a decrease in HER3. These results imply that the blocking dose of antibody (cetuximab or DL3.6b) may be inducing an upregulation of either receptor in an attempt to maintain proliferation.

### ***Ex vivo*** competitive binding assay

**Figure 5:**
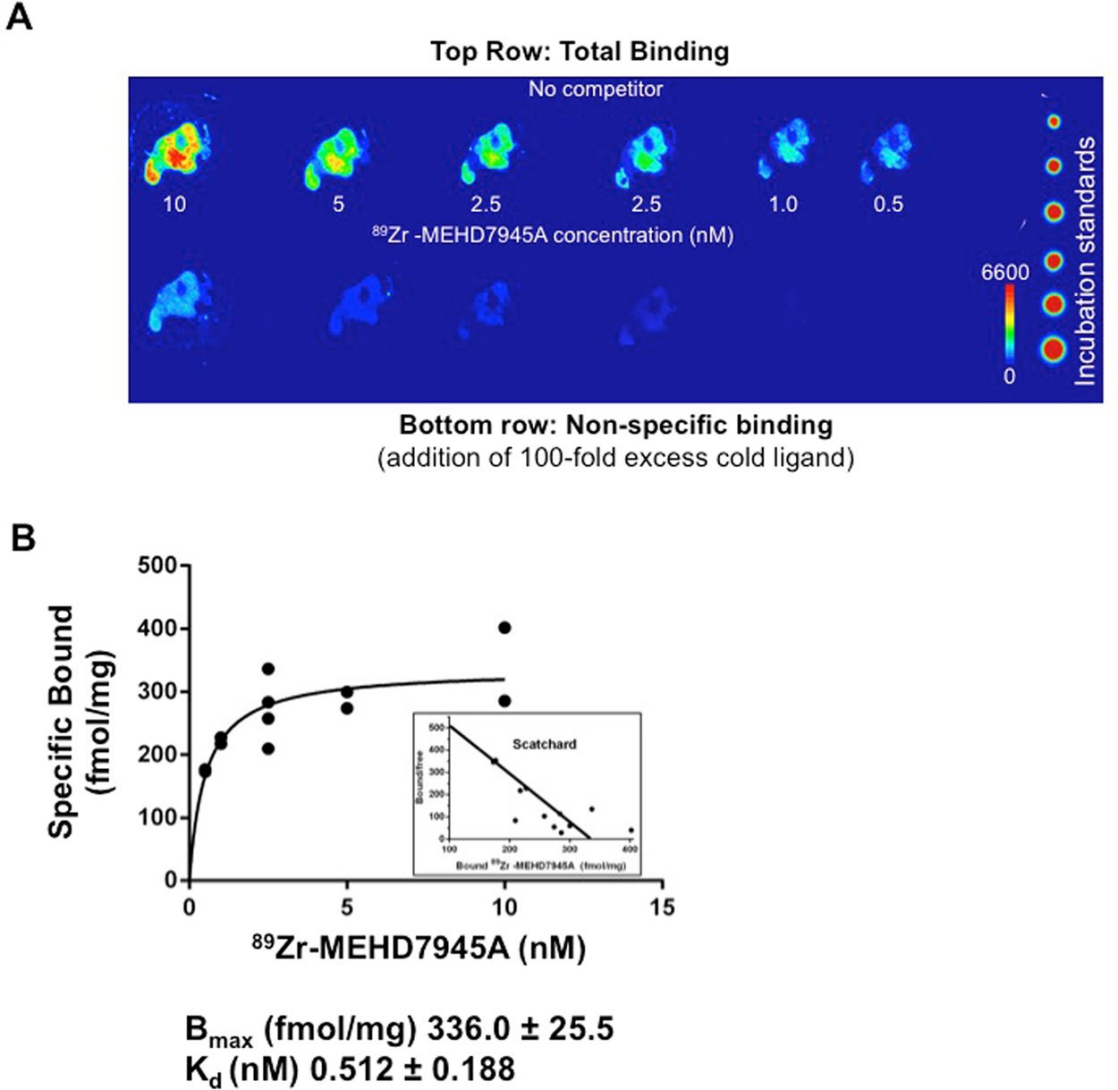
*Ex vivo* competitive binding assay. A digital autoradiograph of AsPC-1 tumor sections displayed saturable, concentration-dependent binding of ^89^Zr-MEHD7945A upon addition of 100-fold excess cold MEHD7945A. (**A**) A non-linear regression analysis and Scatchard plot of ^89^Zr-MEHD7945A plotted against the amount of bound ligand shows the Bmax ~ 336 fmol/mg and a K_D_ ~ 0.51 nM (**B**).

Figure 5A showed that an addition of excess cold MEHD7945A (100 nM) blocks the binding of ^89^Zr-MEHD7945A (1 nM). This demonstrated a saturable, concentration-dependent binding of the tracer. From the Scatchard plot (Fig. 5B), the B_max_ was found to be 336.0 ± 25.5 fmol/mg or 2 × 10^5^ binding sites per cell (assuming 1 million cells per tumor) and the K_D_ was found to be ~0.51 ± 0.12 nM.

## Discussion

The MEGHAN trial was a randomized phase II study that evaluated drug efficacy of MEHD7945A compared to cetuximab in patients with recurrent/metastatic squamous cell carcinoma of the head and neck that progressed after chemotherapy^26^. Compared to cetuximab treatment alone, the study found that MEHD7945A did not significantly improve patient outcomes, even those with amplified neuregulin-1 expression, a HER3 ligand, as measured by disease free survival^26^. Despite this dismal outcome, the combined blockade of EGFR and HER3 merits a second look with trials that particularly focus on EGFR treatment-resistant patient populations. This rationale stems from an overwhelming body of evidence showing that dual inhibition of EGFR and HER3 promotes desensitization of lesions to EGFR blockade through HER3 crosstalk inhibition^27–29^. With this perspective, our initiative to develop a companion diagnostic to MEHD7945A is potentially useful for selecting patients within this space in order to benefit from this treatment. To the best of our knowledge, we are the first to develop a new PET radiotracer that simultaneously delineates both of EGFR and HER3 receptors. Only one recent study has described a bi-specific immunoPET imaging tracer, albeit, of different receptors, CD3 and EpCAM^30^. Other previous and currently investigated immunoPET tracers specific to these two receptor tyrosine kinases are limited to single antigen detection with either EGFR^25,31–33^ or HER3^34–37^ alone. The paradigm is shifting, however, to combinatorial drug cocktails to debulk tumor. The quest for a potent and efficient therapy led to the development of bispecific antibodies or fragments to target more than one molecular signature (tumor associated antigens) amplified in lesions, e.g., EGFR/IGF1-R^38^, EGFR/Met^39^, and HER2/HER3^40^. This stems from the rationale that a single agent targeting multiple markers vis-à-vis a monospecific approach can strongly stabilize specificity and selectivity for tumor instead of normal tissues. To select a patient population who stands to benefit from the treatment, non-invasive and quantitative imaging biomarkers are of utmost need. We believe that ^89^Zr-MEHD7945A satisfies this initiative.

The low expression of HER3 in malignancies and moderate expression in normal tissues^41^ mandate targeted drugs and imaging radiotracers to have a high avidity to this antigen^40^. Sub-nanomolar or higher affinities are preferable for improved tumor specificity and selectivity^36,40^. We examined whether the binding affinity of ^89^Zr-MEHD7945A for EGFR and HER3 were retained after radiolabeling. The results from the *in vitro* and *ex vivo* competitive binding assays exhibited nanomolar affinities, in good agreement with the established K_D_ of the unmodified antibody^17^. Other immunoPET tracers singly targeting HER3 fall within the same range (i.e. 2.7 nM^42^ and 6.8 nM^37^). Only one other tracer, the affibody-based probe ^68^Ga-HEHEHE-Z08698-NOTA, exhibited picomolar binding affinity (50 pM)^36,43^. Established affinities of other immunoPET EGFR tracers are within the same range. For example, an affibody labeled with F-18 displayed a K_D_ ~ 37 nM whereas a ^67^Ga-DOTA-cetuximab F(ab′)2 reported a K_D_ ~ 8.6 nM, making the K_D_ for our radiotracer within reasonable range.

The acquired PET images are validated by the tissue distribution data. Radiotracer uptake was observed in the lung, liver, spleen and bone, which can be attributed to the moderate expression of both EGFR and HER3 and their role in tissue growth^44^. This is supported by the data from competitive blocking studies where the tissues (listed above, excluding bone) in the blocked cohort of mice had lower probe accumulation at 48 h than the unblocked cohort (Fig. 2E, Supplementary Table S3). Cross-reactivity of the fully human MEHD7945A with murine EGFR and HER3 is expected; murine EGFR is 94% homologous to human EGFR^45^ whereas murine HER3 is orthologous to the human receptor with 90.8% sequence homology^46^. Bone accumulation can be a combination of specific EGFR/HER3 targeted delivery with reported expression of both RTKs in skeletal tissues^47,48^ and/or non-specific deposit of demetalated ^89^Zr from its chelate DFO. The latter is evidenced by the free ^89^Zr observed as early as 24 h with 95–97% of the tracer remaining intact at 120 h (Supplementary Fig. S1). The competitive inhibition experiment of ^89^Zr-MEHD7945A using unmodified MEHD7945A together with non-specific IgG isotopic probe control from our data is evident of specific tumor targeting of ^89^Zr-MEHD7945A and thereby eliminating the enhanced permeation effect as the underlying mechanism of delivery. Co-localization of the tracer with EGFR/HER3 expression as shown in the autoradiography and IHC studies further proved the probe’s selectivity for the receptors.

Despite the high number of EGFR and HER3 present as verified through B_max_ values, flow cytometry and immunoblots, the tumor accumulation of the probe in AsPC-1 xenografts uptake was lower compared to BxPC-3 models. With PDAC reportedly possessing dense stroma, we initially rationalized that high levels of collagen, an essential component of the extracellular matrix may perturb tumor penetration of the probe. The trichome stain (Supplementary Fig. S4C), however, did not display any significant disparity in collagen levels between the two types of tumors. Looking at cell density from a pathological perspective, the BxPC-3 tumors were denser than the AsPC-1 tumors as shown by cell count (Supplementary Fig. S4D). This consequently lowered receptor sites available, which potentially explains the lower PET volume of interest (VOI) uptake attained in the AsPC-1 tumors.

We interrogated the dual specificity of the probe for either EGFR or HER3 through competitive binding assays. Interestingly, blocking either EGFR or HER3 separately increased or sustained the binding levels of the probe compared to blocking both receptors simultaneously. The highest blocking doses administered can be considered a treatment dose (HER3 IC_50_ ~ 0.12 µg/mL and EGFR IC_50_ ~ 0.02 µg/mL for the *in vitro* study^17^, and 0.04–1 mg per mouse *in vivo*^49,50^), potentially eliciting an activated feedback loop. Results of the study seemingly imply an “EGFR-only” mechanism of probe uptake. Looking closely at both AsPC-1 and BxPC-3 control untreated cells, the flow cytometry data indicated very low HER3 expression compared to EGFR, which is five-fold higher (Supplementry Table S1). We believe that any marginal change in HER3 when blocked with DL3.6b combined with the magnitude of EGFR expression may be below the sensitivity threshold of the radiotracer. With the *in vivo* competition assays, it would seem like the blocks were unsuccessful with the tracer uptake between control and blocked groups rendered statistically insignificant. In this case, external validation with immunohistochemistry (Fig. 4C) and western blots (Fig. 4D) supported feedback compensation of either EGFR or HER3, albeit on a smaller scale. We must keep in mind that the tumor was exposed to the blocking antibodies acutely vs. chronic exposure in a therapeutic setting; thus, a sustained and higher HER3 induction may not have been achieved. Nevertheless, the tracer detected varied expression of either RTKs. Admittedly, one of the main limitations of the probe lies in identification of which receptor is upregulated.

Our previous work demonstrated that the probe detected solely HER3 feedback upon AKT inhibition in triple negative breast cancer^51^. Moreover, we have unpublished data showing similar HER3 detection by the radiotracer in colorectal cancer upon MEK treatment. Our findings were corroborated by a wide body of evidence demonstrating HER3 feedback upon independent blockade of EGFR in colon cancer^12^, head and neck small cell cancer^13^, lung cancer^52^ and triple negative breast cancer. Thus, simultaneous blockade of both EGFR and HER3 with MEHD7945A is potentially more efficacious than single agent monotherapy.

## Conclusion

In summary, we have successfully developed an EGFR/HER3 targeting immunoPET companion diagnostic to MEHD7945A (duligotuzumab) and evaluated its properties in the preclinical setting. This probe has high potential to non-invasively delineate and stage EGFR and HER3 positive tumors with high affinity and selectivity.

## Methods

### Validation of EGFR and HER3 receptor expression in pancreatic cancer cells

Validation of EGFR and HER3 expression in AsPC-1, BxPC-3 and Mia PACA2 pancreas cancer cells was conducted via standard flow cytometry and immunoblot analysis. Cells were labeled with anti-EGFR-AF488 (AY13, Biolegend) and anti-HER3-APC (1B4C3, Biolegend) and analyzed for receptor expression using BD LSR II FLOW cytometer (BD Biosciences).

### Western Blotting

The EGFR and HER3 protein expression were determined by SDS-PAGE using the Invitrogen XCell SureLock system. Briefly, proteins were extracted from cell pellets with RIPA buffer and protease and phosphatase inhibitors (HALT, ThermoFisher). Fifteen micrograms of protein were resolved by 4–12% SDS-PAGE and transferred to membrane. After blocking with 5% milk in TBS-0.1% Tween-20, membrane was incubated with anti-EGFR-XP (D38B1, Cell Signaling), anti-HER3-XP (D22C5, Cell Signaling), or anti-β-Actin (mAbcam 8226, Abcam) antibody overnight at 4 °C. After incubation with secondary antibody (anti-mouse HRP or anti-rabbit HRP, Amersham) the membrane was visualized by Ammersham ECL (GE Life sciences) and read using a ChemiDoc imaging system (Bio-Rad) and analyzed using image lab touch software 2.2 (Bio-Rad).

### Radiolabeling of MEHD7945A

p-Benzyl-isothiocyanate-desferrioxamine (DFO-Bz-SCN, Macrocylics, Inc.) was conjugated to MEHD7945A and a non-specific human IgG isotype (Sigma) according to published protocols^53,54^. The synthesis was performed using 7:1 and 5:1 mole equivalence of DFO-Bz-SCN to MEHD7945A or IgG, respectively in a 0.9% saline, pH ~9 at 37 °C for 1 h. The pure mAb DFO-conjugates were obtained through by purification with a spin column filter with a molecular weight cut-off of 30 kDa (GE Vivaspin 500) to remove unbound chelate.

^89^Zr-oxalate was produced as previously described^55^. Approximately 1 mCi (37 MBq) of ^89^Zr-oxalate was neutralized to pH 7.0–7.2 using 1 M NaOH. MEHD7945A-DFO (200 µg) was added to the ^89^Zr solution. The reaction was quenched after 1–1.5 h incubation at room temperature upon addition of 10 µL of 50 mM EDTA (pH~7) to eliminate any non-specifically bound ^89^Zr. Radiolabeling efficiency and purity were determined via radio-instant Thin Layer Chromatography (iTLC) using silica gel-impregnated iTLC strip (Agilent Technologies, Santa Clara, CA) and 50 mM EDTA as the solid and mobile phase respectively. Pure ^89^Zr-MEHD7945A was obtained by passing through a spin column centrifugation filter (GE Vivaspin 500, MWCO: 30 kDa) with saline as the diluent. A radiochemical purity of >99% was achieved based on iTLC analysis. ^89^Zr-MEHD7945A was assessed for immunoreactivity as previously described^56^. Demetallation of ^89^Zr was monitored in 0.9% saline and 1:1 human serum:saline over time at 37 °C via iTLC.

### Cell Lines and Small Animal Xenografts

All animal handling, manipulations, and experiments were conducted in accordance with the guidelines and regulations set by Wayne State University Institutional Animal Use and Care Committee (IACUC), MSKCC Animal Care and Use Committee and Research Animal Resource Center, which are accredited by the Association for Assessment and Accreditation of Laboratory Animal Care (AAALAC). For imaging experiments, female SCID mice (6–8 week old, Taconic) were subcutaneously implanted on the shoulders with BxPC-3 and AsPC-1 pancreatic cancer cells that were cultured in RPMI 1640 + 10% FBS + 1% NEAA + 1% PenStrep. All cells (5 × 10^6^ cells/tumor) in 150 µL 1:1 media:Matrigel (BD Biosciences, Bedford, MA) were injected on the right shoulder. Monitoring of tumor growth was performed weekly with calipers. The tumor volume was calculated using the formula: length × width × height × π/6. Mice with tumor volumes ranging from 150–250 mm^3^ were utilized. For *in vitro* experiments, Mia PACA2 cells were cultured in DMEM + 10% FBS + 2.5% Horse Serum + 1% PenStrep. All cells were maintained at 37 °C at a 5% CO_2_ atmosphere.

### Internalization Assay

Internalization of ^89^Zr-MEHD7945A was evaluated on AsPC-1, BxPC-3 and MIA PACA2 pancreatic cancer cell lines. Wells were seeded with ~1 × 10^5^ cells and incubated overnight. Radiolabeled protein [1 μCi/mL (37 kBq/mL, 20 μg)] in 1 mL of media was added to each well. The plates were incubated at either 37 °C or 4 °C for 0.5–24 h. Following each incubation period, the media was collected and the cells were rinsed with 1 mL 1× phosphate buffered saline (PBS) twice. Surface-bound activity was removed by washing the cells in 1 mL 100 mM acetic acid + 100 mM glycine (1:1, pH 3.5) at 4 °C. The cells were then lysed with 1 mL 1 M NaOH. All washes (media plus PBS, acid and alkaline) were collected in separate tubes and measured for counts using a gamma counter (Perkin Elmer). The %-internalized activity was calculated as the ratio of the activity of the lysate and the total activity collected from the media plus PBS wash, acid, and base washes.

### Determination of binding affinity (K_D_) of MEHD7945A-DFO and ^89^Zr-MEHD7945A

Surface plasmon resonance (SPR) (Biacore 3000, GE Healthcare) evaluated the binding affinity expressed as the equilibrium binding constant, K_D_ of the MEHD7945A-DFO and the unmodified mAb using previously published protocols^57^. Immobilization of HER3 and EGFR proteins on a CM5 sensor chip was performed at 25 °C by amine-coupling chemistry using Biacore 3000 (GE Healthcare). To determine kinetic rate constants, the processed data were fitted to 1:1 Langmuir binding model using BIAevaluation software.

The total EGFR and HER3 binding sites on AsPC-1 and BxPC-3 cell lines were determined by homologous competitive saturation binding assay using radiolabeled ^89^Zr-MEHD7945A. Briefly, after detachment from cell culturing flasks by trypsinization, AsPC-1 and BxPC-3 were collected and re-suspended in PBS/1% BSA. Radiolabeled ^89^Zr-MEHD7945A was incubated with increasing concentration of MEDH7945A to AsPC-1 or BxPC-3 cells in PBS/1% BSA at room temperature. Sample incubation was terminated by vacuum filtration of samples through glass microfiber filters followed by triple PBS/1% BSA wash and counted on Wizard2 gamma counter. The IC_50_ was analyzed by sigmoidal dose response curve using a four-parameter logistic nonlinear regression. Two-site, non-linear saturation binding model was employed to determine K_DHi_ and K_DLo,_ the radioligand concentrations required to achieve a half-maximum binding at equilibrium. The Bmax_Hi_ and Bmax_Lo,_, the maximum specific bindings to the two receptor sites were also analyzed.

### *In vitro* competitive binding assay

AsPC-1 and BxPC-3 cells were plated at 200,000 cells/well and allowed to adhere overnight at 37 °C with 5% CO_2_. The cells were then co-incubated with either 10× (1 μg) or 25×, (2.5 μg) cold MEHD7945A, cetuximab, or DL3.6B and ^89^Zr-MEHD7945A [~0.5 μCi (0.0185 MBq), 100–125 ng] in 1 mL of complete media for 1 h at 37 °C with 5% CO_2._ Following incubation, the media was collected and the cells were rinsed with 1 mL 1× PBS twice. The cells were then lysed with 1 mL 1 M NaOH. All washes (media plus PBS, and alkaline) were collected in separate tubes and measured for counts using a gamma counter (Perkin Elmer). The %-bound activity was calculated as the ratio of the activity of the lysate and the total activity collected from the media, PBS, and base washes.

For flow cytometry studies, AsPC-1 and BxPC-3 cells were prepared as described above and then incubated with 10× or 25× cold antibodies in complete medium for 48 h at 37 °C with 5% CO_2_. After incubation, media was removed and cells were washed with 1× PBS and subsequently labeled with anti-EGFR-AF488 (AY13, Biolegend) and anti-HER3-APC (1B4C3, Biolegend) and analyzed for receptor expression using BD LSR II FLOW cytometer (BD Biosciences).

### PET Imaging Experiments

^89^Zr-MEHD7945A [200–275 μCi (7.4–10.2 MBq), 44–61 µg, 290–400 pmol] in sterile saline was intravenously (i.v.) administered on the lateral tail-vein in mice (n = 3–4) bearing either BxPC-3 or AsPC-1 s.c. xenografts. Small-animal PET scans were acquired between 24–96 h post-tracer administration using microPET-R4 and/or Focus 120 scanners (Concorde Microsystems). The mice were fully anesthetized with 1–2% isoflurane (Baxter, Deerfield, IL) throughout the scan. Images were reconstructed via filter back projection. ASIPro VM^TM^ software (Siemens Concorde Microsystems) was used to analyze volumes-of-interest (VOI) on various planar sections from the acquired image by manually drawing on the tumor site and on select organs. The average VOI was calculated and expressed as % injected dose per gram of tissue (%ID/g). To prove specificity, competitive inhibition studies were conducted by co-administering ~200–500 µg (1.33 nmol–3.33 nmol) of non-radioactive MEHD7945A in BxPC-3 tumor-bearing mice (n = 3–5). PET imaging with ^89^Zr-IgG [230–250 μCi (8.51–9.25- MBq, 306–336 pmol, 46–50 μg) was conducted in mice with BxPC-3 and AsPC-1 tumors to assess non-specific accumulation of the tracer.

### Tissue distribution and *in vivo* competitive specificity

The tissue distribution of ^89^Zr-MEHD7945A was assessed in mice-bearing BxPC-3 tumors. A tracer dose of 10–15 μCi (370–555 kBq, 1–2 µg, 6.7–13.3 pmol) was injected i.v. on the lateral tail vein. *In vivo* competitive specificity assays were conducted in separate cohorts (n = 4–5 per group) of BxPC-3 and AsPC-1 xenografts. The tracer dose 25–30 μCi (0.925–1.11 MBq, 5–6 µg, 33.3–40 pmol) was co-injected with 10-fold higher blocking doses of either MEHD7945A (50 μg), cetuximab (50 μg) or the anti-HER3 mAb, DL3.6B (50 ug). Euthanasia via CO_2_ asphyxiation was performed between 24–120 h post injection (p.i.) of the tracer. For the *in vivo* competitive binding assay, mice were sacrificed 48 h p.i. Blood was immediately collected via cardiac puncture. Select organs were harvested, rinsed and dried to remove excess water. Bound activity was measured using a gamma counter. Activity measurements were background- and decay-corrected to the time of counting. The tissue uptake, expressed as % injected dose per gram of tissue was calculated. Western blots and immunohistochemistry (IHC) validated the tumor uptake of the tracer.

### Autoradiography and immunohistochemistry

Following PET imaging, tumors were excised, embedded in optimal-cutting-temperature mounting medium (OCT, Sakura Finetek), frozen on dry ice and series of 10 μm frozen sections cut. To determine radiotracer distribution, digital autoradiography was performed by placing tissue sections in a film cassette against a phosphor imaging plate (Fujifilm BAS-MS2325; Fuji Photo Film) at −20 °C for an appropriate exposure period. Phosphor imaging plates were read at a pixel resolution of 25 μm with a Typhoon 7000 IP plate reader (GE Healthcare). Contiguous frozen sections were then used for staining and microscopy. Sections were fixed with ice-cold acetone for 10 minutes and dried at room temperature for 10 minutes before re-hydration in tap water followed by PBS. Superblock (ThermoFisher) was used to block slides for 40 minutes at room temperature. Slides were incubated in primary antibodies HER3-XP (D22C5, Cell Signaling, 1:40) and EGFR-XP (D38B1, Cell Signaling, 1:50) overnight at 4 °C. Slides were developed using Pollink-2 Plus HRP rabbit with DAB (GBI Labs, D39–18) or Klear mouse HRP with DAB (GBI Labs, D52–18) and dehydrated with alcohols and xylenes before being covered with permount. Imaging was performed with a Nikon Eclipse Ci microscope (Nikon) using Spot Basic 5.2 software (Diagnostic Instruments, Inc). Images were analyzed in panoramic viewer 1.15.4 for windows (3D HisTech)^58^. IHC evaluation and scoring for EGFR^59^ and HER3^60^ followed previously described protocols^60^.

### *Ex vivo* competitive binding studies

Sequential 10 μm frozen sections cut from a fresh-frozen AsPC-1 tumor were incubated in binding buffer (0.1%BSA, 40 mg/L bacitracin in PBS, pH 7.4) containing 0.5–10 nM ^89^Zr-MEHD7945A for 1 h at room temperature in a humidified chamber. Non-specific binding was assessed on additional sections by the addition of 100-fold mole excess of cold MEHD7945A. Activity standards of each concentration with a known volume were spotted onto a flexible TLC sheet (Avantor Performance Materials, Center Valley, PA). Sections and standards were then exposed to a storage phosphor plate, and read as described above. Activity concentrations of whole tumor section areas and standards were determined using ImageQuant 7.0 software, and subsequently converted to a molar concentration using the tumor sectional area calculated from the image, multiplied by the 10 µm section thickness to determine volume. Binding parameters (B_max_, K_D_) were determined by non-linear regression in addition to Scatchard analysis.

### Statistical analysis

Statistical analysis was performed using two-way ANOVA test in *in vitro* assays and tumor uptake comparison unless otherwise stated. A value of *P* < 0.05 was considered statistically significant. Data were expressed as the mean ± S.D. All analyses were performed using GraphPad PRISM v.6 software unless otherwise stated.

### Data availability

All data generated or analyzed during this study are included in this published article (and its Supplementary Information files). The datasets generated during and/or analyzed during the current study are available from the corresponding author on reasonable request.

### Ethical approval

The Wayne State University Institutional Animal Use and Care Committee (IACUC) approved all animal experiments during an ethical review. Principles of laboratory animal care were followed and all procedures were conducted according to guidelines established by the National Institutes of Health, and every effort was made to minimize suffering.

## Electronic supplementary material

Supplementary Figures and Tables
