# Supplementary material for: Imaging EGFR and HER3 through 89Zr-labeled MEHD7945A (Duligotuzumab)
Source: Sci Rep. 2018 Jun 13;8:9043. doi: 10.1038/s41598-018-27454-6 (PMC5998059; doi:10.1038/s41598-018-27454-6)
Supplement: Supplementary file 1 — Supplementary Figures and Tables [file 41598_2018_27454_MOESM1_ESM.pdf]

Imaging EGFR and HER3 through ^{89}Zr-labeled MEHD7945A (Duligotuzumab)

Brooke N. McKnight[1], Akhila N. W. Kuda-Wedagedara[1], Kuntal K. Sevak[2], Dalya Abdel-Atti[2], Wendy N. Wiesend[3], Anson Ku[2], Dakshnamurthy Selvakumar[1], Sean D. Carlin[2], Jason S. Lewis[2,4,5] Nerissa T. Viola-Villegas[1,2*]

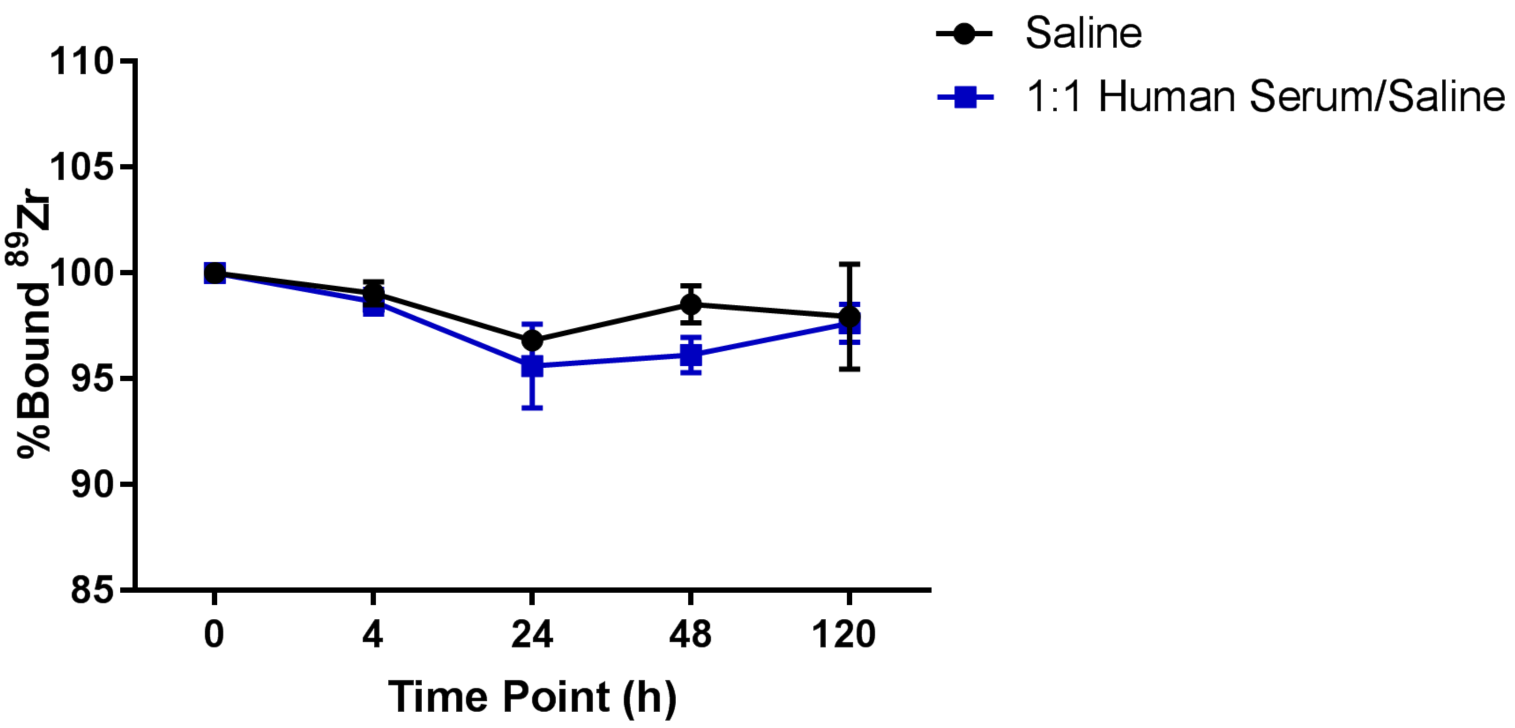

Time point (h)	Saline (Mean %Bound ± S.D.)	1:1 Human Serum:Saline (Mean %Bound ± S.D.)
**4**	99.05 ± 0.54	98.65 ± 0.60
**24**	96.82 ± 0.41	95.62 ± 1.99
**48**	98.52 ± 0.89	96.14 ± 0.83
**72**	95.39 ± 1.02	96.92 ± 2.80
**120**	97.95 ± 2.48	97.64 ± 0.90

Supplementary Figure S1. Stability of ^{89}Zr-MEHD7945A in serum and saline up to 120 h. ^{89}Zr-MEHD7945A was incubated in saline or 1:1 human serum:saline for up to 120 h and demetalation of ^{89}Zr was measured via iTLC.

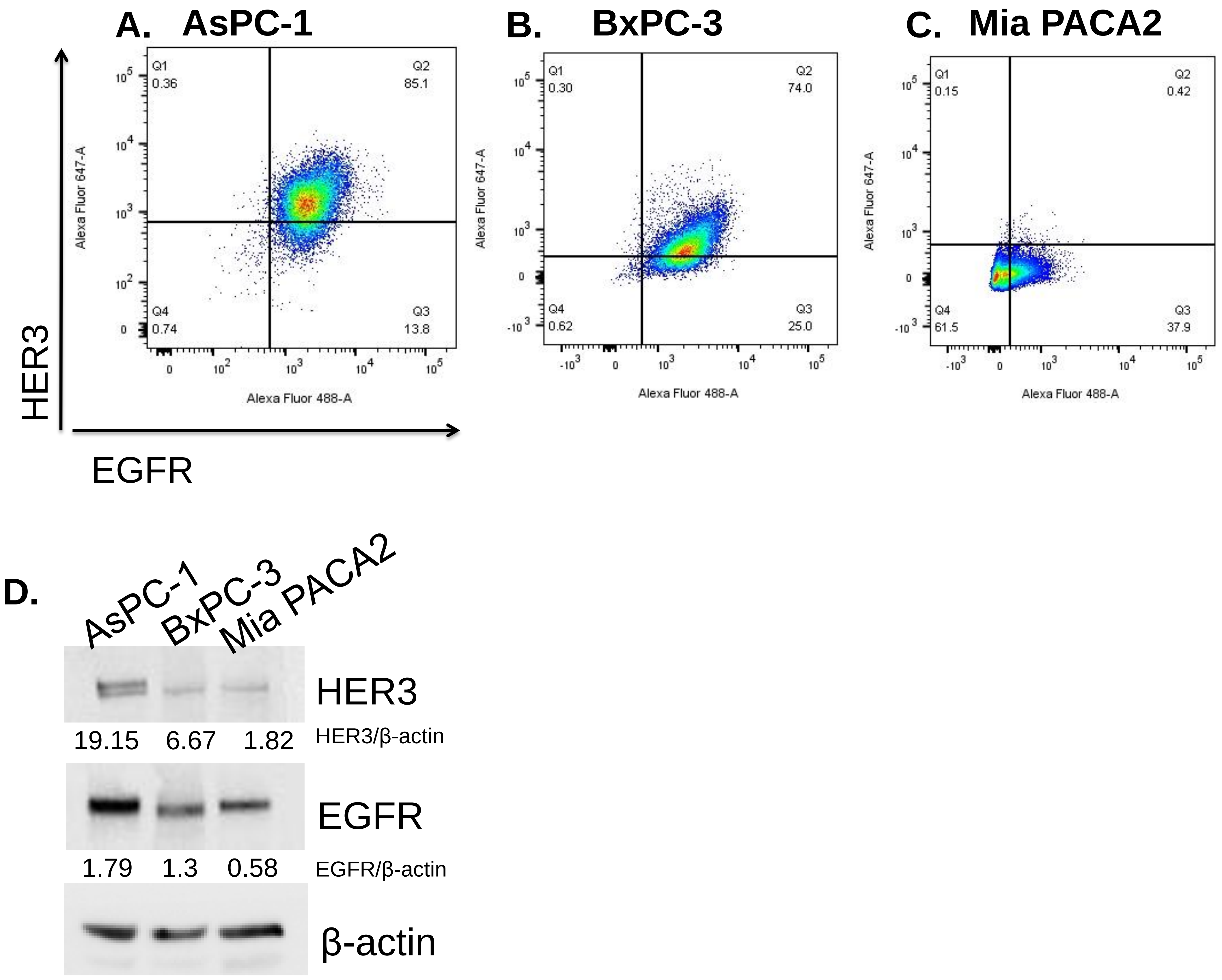

Supplementary Figure S2. EGFR and HER3 expression on cell lines. Flow cytometry demonstrates expression of EGFR and HER3 in AsPC-1 **(A)**, BxPC-3 **(B)**, and Mia PACA2 **(C)**. Western blot analysis exhibits total HER3 and EGFR protein on AsPC-1, BxPC-3, and Mia PACA2 cell lysates **(D)** Densitometry is displayed as a ratio of target protein to loading control β-actin.

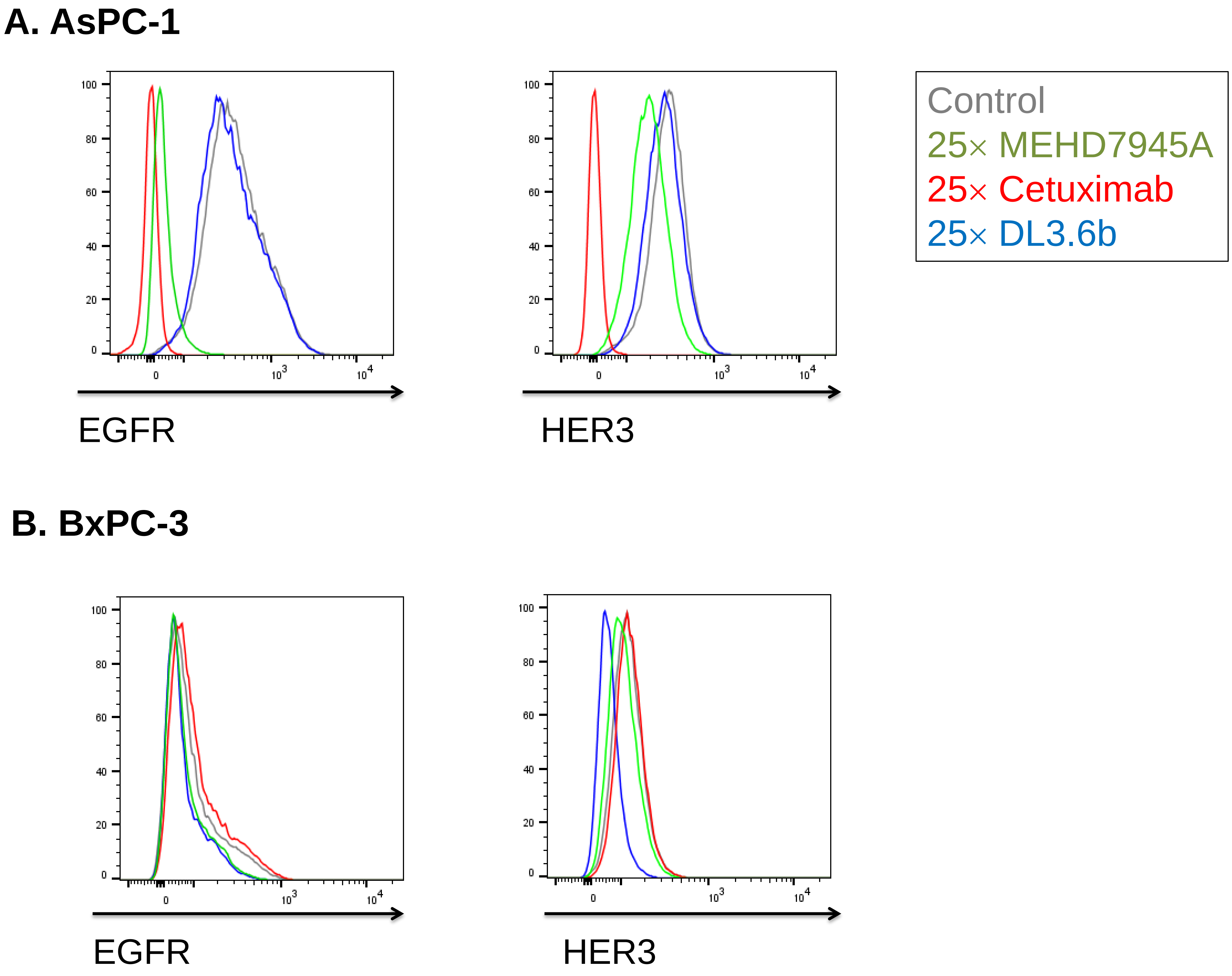

Supplementary Figure S3. Flow cytometry analysis of EGFR and HER3 receptor expression after blocking. AsPC-1 **(A)** and BxPC-3 **(B)** cells incubated with 25-fold cold doses of MEHD7945A (green), Cetuximab (red) and DL3.6b (blue) showed a decrease in cell populations possessing both EGFR (**left**) and HER3 (**right**) expression compared to unblocked cells (gray). Expression on the x-axis is expressed as median fluorescent intensity (MFI).

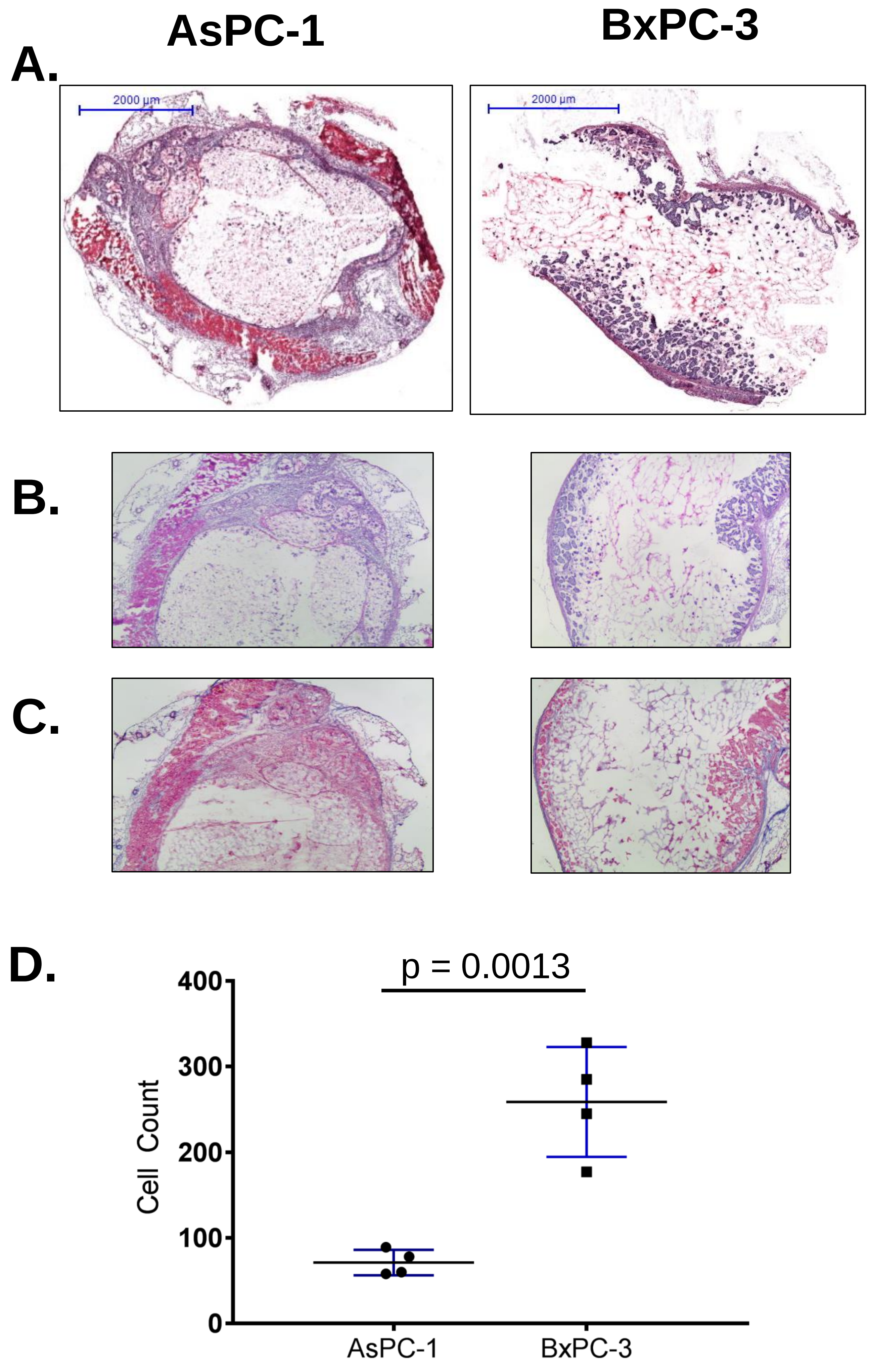

Supplementary Figure S4. Immunohistochemistry. AsPC-1 (left) and BxPC-3 (right) tumors showed viability based on full sized H&E (**A**), 20× magnified H&E (**B**), and 20× trichrome stain **(C**). Higher cell density was observed in BxPC-3 vs. AsPC-1 (258 ± 64 vs. 71 ± 15, p = 0.0013) (**D**).

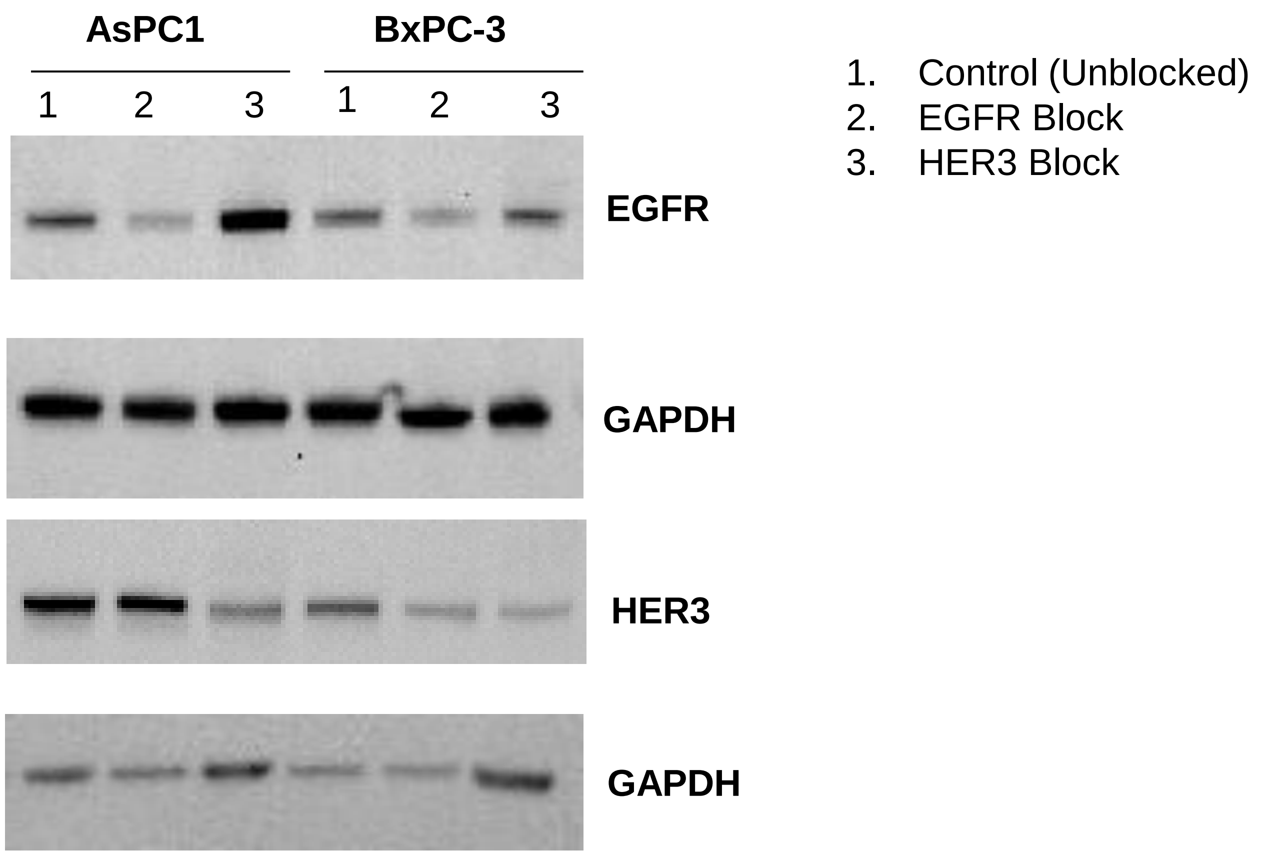

Supplementary Figure S5. Western blots of AsPC-1 (left) and BxPC-3 (right) tumors demonstrating EGFR and HER3 total protein in control untreated (lane 1), cetuximab-blocked (lane 2) and HER3-blocked (lane 3) cohorts of mice

Supplementary Table S1. In vitro competitive binding with MEHD7945A, cetuximab and DL3.6b exhibited different uptake of ^{89}Zr-MEHD7945A in AsPC-1 and BxPC-3 cells. Flow cytometry data expressed as mean fluorescent intensities (MFI) in both cell lines exposed to 25× MEHD7945A, cetuximab and DL3.6b display varied EGFR and HER3 expression.

	AsPC-1						BxPC-3					
	^{89}Zr-MEHD7945A Binding (Mean ± S.D.)	P	EGFR (Mean MFI ± S.D.)	P	HER3 (Mean MFI ± S.D.)	P	^{89}Zr-MEHD7945A Binding (Mean ± S.D.)	P	EGFR (Mean MFI ± S.D.)	P	HER3 (Mean MFI ± S.D.)	P
**Control (No Block)**	5.55 ± 0.64		340.3 ± 32.8		79.6 ± 11.5		11.72 ± 3.54		307.7 ± 11.2		127.0 ± 3.6	
**25x MEHD7945A**	1.06 ± 0.82	0.0001	32.6 ± 2.8	0.0001	57.3 ± 3.7	0.0962	0.29 ± 0.16	0.0001	182.7 ± 28.4	0.0001	95.4 ± 7.7	0.0123
**10x MEHD7945A**	1.97 ± 0.74	0.0001					1.02 ± 0.34	0.0001				
**25x Cetuximab**	1.47 ± 0.42	0.0001	0.10 ± 0.9	0.0001	80.4 ± 6.9	0.9996	1.02 ± 0.23	0.0001	3.3 ± 0.8	0.0001	130.3 ± 4.0	0.9774
**10x Cetuximab**	1.09 ± 0.47	0.0001					1.21 ± 0.46	0.0001				
**25x DL3.6b**	5.60 ± 0.51	n.s.	336.0 ± 3.6	0.0001	48.2 ± 4.9	0.0128	16.36 ± 0.88	0.0001	284.0 ± 13.1	0.0739	63.3 ± 8.2	0.0001
**10x DL3.6b**	3.47 ± 0.26	0.0194	.				6.98 ± 0.69	0.0001				

Supplementary Table S2. Tissue uptake (Mean %ID/g ± S.D.) of ^{89}Zr-MEHD7945A in BxPC-3 tumor xenografts.

Tissue	24 h	48 h	96 h	120 h	48 h Block
Blood	10.49 ± 0.89	8.37 ± 0.60	5.32 ± 1.04	2.99 ± 0.54	10.24 ± 0.44
Tumor	18.97 ± 9.13	31.08 ± 3.89	32.85 ± 4.70	32.73 ± 5.31	12.97 ± 3.44
Heart	3.96 ± 0.98	2.92 ± 0.59	2.13 ± 0.81	1.23 ± 0.32	4.13 ± 0.37
Lungs	4.97 ± 2.00	4.49 ± 2.85	5.88 ± 1.64	2.85 ± 1.31	7.77 ± 1.84
Liver	8.69 ± 3.96	9.75 ± 1.98	5.79 ± 2.26	7.62 ± 0.68	2.99 ± 1.72
Spleen	4.34 ± 0.30	3.72 ± 1.77	4.26 ± 1.39	2.68 ± 1.42	1.49 ± 0.60
Stomach	1.73 ± 0.81	1.30 ± 0.69	1.17 ± 0.42	1.18 ± 0.27	0.96 ± 0.60
Gut	0.99 ± 0.23	1.17 ± 0.31	0.78 ± 0.32	0.73 ± 0.16	0.70 ± 0.07
Pancreas	1.71 ± 1.95	1.06 ± 0.39	1.38 ± 0.61	0.96 ± 0.20	0.97 ± 0.75
Kidneys	2.26 ± 1.52	2.39 ± 0.41	2.95 ± 1.12	2.31 ± 0.55	2.90 ± 1.08
Bone	5.05 ± 1.82	6.84 ± 3.85	8.09 ± 3.85	8.86 ± 4.34	4.03 ± 1.60
Muscle	0.32 ± 0.07	0.38 ± 0.19	0.46 ± 0.29	0.30 ± 0.18	0.64 ± 0.52

Supplementary Table S3. Tumor uptake (Mean %ID/g ± S.D.) of ^{89}Zr-MEHD7945A in AsPC-1 and BxPC-3 tumor xenografts blocked with Cetuximab or DL3.6b.

Tumor	Control	DL3.6b Block			Cetuximab Block		
**AsPC-1**	19.30 ± 5.59	18.79	±	8.75	7.72	±	6.70
**BxPC-3**	28.56 ± 10.81	46.89	±	20.44	21.79	±	14.19
